# Navigating the unchartable: paths to promotion and tenure in health professions education

**DOI:** 10.1007/s40037-016-0306-0

**Published:** 2016-10-20

**Authors:** Joanna Bates, Brett Schrewe

**Affiliations:** 1 Department of Family Practice and Centre for Health Education Scholarship, Faculty of Medicine, University of British Columbia, Vancouver, Canada; 2 Department of Pediatrics, Faculty of Medicine, University of British Columbia, Vancouver, Canada

Varpio et al. lay out challenges that new faculty face in negotiating different meanings of scholarly contribution, teaching and service in a quest for promotion and tenure within their universities [1]. Promotion and tenure processes are indeed a labyrinth for young faculty. The halls are dimly lit, the thread to follow is tenuous and one may have the sense of foreboding that a wrong turn or miscalculated step may put one’s career advancement squarely in the arms of the Minotaur. Searching desperately for a map, young faculty find that there are none. Rather, pathways through this maze shift and change, with review committees moving and adjusting the signposts as times and circumstances demand. Within even a single university community, each unit participates differently in scholarly activities, and similar words in broad university policies come to powerfully connote locally different meanings in individual departments. How can up-and-coming faculty successfully navigate their way?

Navigating this future means first developing a deeper sense of our heritage. Universities first formed in Europe as self-regulating communities of scholars and learners that determined the qualifications of their members. Today, that spirit lives on in the form of communities of tenured peers, who rely upon specific processes and practices in the pursuit of advancing knowledge. Formal definitions of terms that are broadly shared across universities, such as ‘teaching’, ‘research’, and ‘service’, carry a veneer of uniformity, yet their meanings have evolved over time. In turn, local connotations of these terms held by faculties – even within the same institution – are highly particular and contingent upon more immediate assumptions of ‘what matters’. Echoing Wenger-Trayner’s description of communities as ‘landscapes of practice’ [2], engaged participation and useful contributions within universities can look strikingly different depending upon which hallway one’s office is located. Scholarly contribution for a basic scientist connotes peer-reviewed published research; for a fine arts scholar, scholarly contribution may be demonstrated through original interpretations that transform others’ practice both within their discipline and beyond its borders.

The highly particular comes to be embodied in the members of the local community and, in turn, the record of their work. Academic accomplishments may powerfully resonate locally and within one’s discipline, yet in most universities, faculty reviews are conducted at multiple levels: department, faculty and university-wide. As evidenced within institutional documents, policies and procedures, scholarly contribution must be translatable from the unit to the broader university community in order to achieve promotion and tenure. Both of our examples above fit into the broader framework of legitimate university activities, yet a shared sense of these activities is rarely well articulated.

The complexity increases even more when we recognize that our world of Health Professions Education (HPE) is not a well-demarcated discipline; rather, it is a creative interdisciplinary field in which different perspectives and healthy debates flourish. Debate is the lifeblood of academic communities, and the debate about the nature of evidence, proof, truth and knowledge is fundamental to HPE [3]. As a field, practitioners come from different disciplines: education, psychology, sociology, as well as nursing, medicine, pharmacy, and so forth. Social sciences are well represented in the field of HPE, but social science understanding of meaning, truth and even research methods confuse and confound biomedical science faculty [4]. As Regehr points out: “… emphasis on an ‘imperative of proof’ in our dominant research approaches has translated poorly to the domain of education, with a resulting denigration of the domain as ‘soft and ‘unscientific’ …”[5].

While junior faculty can be drawn into and come to share the implicit nuances of terms within their own disciplines, this can be more challenging for those who invest in HPE. Different perspectives and approaches may indeed flourish within the field and lead to a creative intellectual ferment, yet one’s contributions may be greeted with skepticism beyond the borders of this space. Young faculty members in HPE, drawn from distinct disciplines, may stumble in the dark as they integrate into a field positioned academically within faculties of health sciences with their positivist leanings. Scholars must cross out of their disciplinary field in order to enter HPE, and at some point must decide whether to fully embed (and leave their discipline behind), whether to straddle – creating meaning for both HPE and their own discipline – or whether to use HPE as their lab for contributions to their own discipline. This boundary crossing can be fraught and this subtext of leaving disciplines behind is rarely explicit in conversations about promotion and tenure [2]. Young faculty are left to negotiate their professional identities and the meaning of their scholarship through a personal and sometimes difficult journey.

As individual faculty members, we are constrained by the processes of the institutions to which we belong. Those processes differ from one university to the next, as Varpio et al. point out, and so they should. Such processes were designed and crafted by individuals and undergo constant change in interpretation. How scholarship is interpreted has changed in both the long-term (philosophy) and the short term [6]. In turn we ourselves, through our development of connotative meaning of words we must use, can change interpretations of activities. Just as definitions of words within dictionaries evolve over time, so too do connotations of language. Terms used for acceptance by our peers into a community of scholars are shaped by the community itself over time. While young faculty might prefer a well-defined path through the maze of review, one key advantage of the flowing nature of connotative meanings is that they are indeed changeable. For the broad does not just shape the particular; rather, members of local communities – including relative newcomers – both debate, inform and can transform what ‘scholarly contribution’ means more widely.

Promotion and tenure may indeed be an odyssey, and an uncertain one. Indeed, Socrates did not have to contend with this maze, but without tenure he also lacked academic freedom. He irritated everyone in the pursuit of understanding the world, and his road ended abruptly in custody and a cup of hemlock. Even so, he helped lay the groundwork for our heritage. Specifically, one in which we challenge assumptions, confront how we think, and reconsider our assumptive definitions. In contemporary universities, we follow his path when we challenge and reinterpret how we might demonstrate teaching, scholarship, and service to our community of peers. Just as Socrates did for Plato, and as Plato did for Aristotle, we in turn open new pathways through the labyrinth for scholars treading in our footsteps.
